# Microscopy Quality Control in Médecins Sans Frontières Programs in Resource-Limited Settings

**DOI:** 10.1371/journal.pmed.1000206

**Published:** 2010-01-26

**Authors:** Derryck B. Klarkowski, Juan Daniel Orozco

**Affiliations:** Médecins Sans Frontières–Operational Center Amsterdam, Amsterdam, The Netherlands

## Abstract

Derryck Klarkowski and Daniel Orozco describe the Médecins Sans Frontières program for monitoring the quality of microscopy for malaria, pulmonary tuberculosis, and leishmaniasis.

## The Challenge

The international humanitarian medical aid organization Médecins Sans Frontières/Doctors Without Borders (MSF) supports a wide network of medical laboratories in resource-constrained countries. Although MSF has always prioritized quality control (QC) for laboratory testing, prior to 2005 we were constrained by two significant limitations. First the QC workload was unsustainable in many programs, as MSF used the traditional protocol of reexamining 10% of negative slides and all positive slides. This is no longer considered practical [1]–[3]. Second MSF had no system for central data analysis as QC was performed independently at the individual laboratory level without standardized protocols.

In May 2005, MSF Operational Centre Amsterdam (MSF-OCA) developed and implemented a standardized, centrally reporting QC program to monitor the quality of microscopy for malaria, pulmonary tuberculosis (TB), and leishmaniasis. The malaria component of this protocol has been adapted by the World Health Organization (WHO) as the recommended international standard for malaria microscopy QC [4]. Here we present a description of the QC protocol and an analysis over a 3-year period, the latter reflecting how the QC protocol has contributed to improved performance.

## The Protocol

The QC protocol was designed to (1) have a small sample size to be feasible across all settings; (2) enable reliable analysis; (3) monitor both false-positive (FP) and false-negative (FN) results; and (4) be applicable to all microscopy testing.

### Monthly QC Sample

#### Sample size

The MSF-OCA protocol is based on a sample size of ten slides/month/site (for each test), as field experience has demonstrated that this QC workload is sustainable in most settings, and on the premise that it is better to perform less QC well than more QC poorly. A small sample size is also important to avoid overloading the limited capacity of the reference laboratory in many resource-constrained settings. Programs are encouraged to include more QC slides if this can be achieved without compromising the quality of the reexamination.

#### Sample selection and reexamination

In summary, each month for each test: (1) Five weak positive slides are selected randomly from all weak positive slides; or if <5 weak positive slides, then all weak positive slides are selected. (2) Five negative slides are selected randomly from all negative slides; or if <5 negative slides, then all negative slides are selected. (3) If there are <5 weak positive (or negative) slides, then the number of negative (or weak positive) slides is increased to give a total minimum sample size of ten. (4) Strong positive slides are excluded from selection in the QC sample. (5) Laboratories unable to perform QC on a minimum of ten slides are assessed on an individual basis.

Blinded QC slides are reexamined within 4 weeks in the field by either a reference laboratory or an independent skilled laboratory technician.

Weak positive slides are defined as ≤9 trophozoites/acid-fast bacilli (AFB)/10 high power fields. These definitions were consistent across laboratory sites. Postimplementation experience now suggests that the criteria for a weak positive should be reduced to ≤9 trophozoites/AFB/100 high power fields.

### Protocol Reliability

While small sample QC has the important advantage of practicality, maintaining reliable analysis is also essential. To compensate for the small number of QC slides reexamined each month, our QC protocol uses analysis of cumulative data over 4-month periods (i.e., 4 months of data), referred to here as “cohort analysis”. These 4-month cohorts are used to increase the sample size analyzed, and as a compromise between the greater statistical stringency of analyzing a larger number of results over a longer duration (e.g., 12 months) versus the greater immediacy of detecting real-time laboratory performance by analyzing QC over a shorter period.

### False-Positive and False-Negative Analysis

To enable FP analysis on small samples, our protocol uses biased sampling to increase the number of positive slides available for reexamination, and the targeting of weak positive slides to increase discriminatory power.

#### Biased sampling

QC protocols that use a small sample size with random sampling of all slides, such as lot quality assurance sampling (LQAS) [2], have the potential disadvantage of being unable to adequately monitor false positivity because of insufficient positive slides at low prevalence rates if QC results are analyzed over short periods of time. To address this, the MSF-OCA protocol uses a biased QC sample of an equal number (whenever possible) of weak positive and negative slides to enable both FP and FN analysis.

#### Targeting weak positives

The protocol selects only weak positive slides because errors of false positivity are most likely to occur during routine microscopy through microscopists reporting negative findings as weakly positive (to be “on the safe side”) [5], or through the misidentification of artifacts as parasites [1]. Using weak positive slides also has greater discriminatory power than reexamining strongly positive slides [1],[6],[7].

However, because FP results are more likely to occur among weak positive slides, reexamining only weak positive slides (rather than all positive slides) may overestimate the FP frequency in routine microscopy. We correct for this by using the formula:
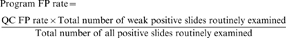
A limitation of this correction is that it assumes a negligible FP frequency for strong positive slides.

### Common Protocol for All Microscopy

A primary objective for MSF-OCA was to develop a protocol that could be used for all microscopy testing. Although LQAS is recommended by WHO and others for AFB direct-smear TB analysis [3], we found this methodology unsuitable for malaria microscopy because determining the LQAS sample size is problematic when there is seasonal variation in the positivity rate. Our protocol therefore uses a fixed rather than variable number of QC slides.

### Laboratory Performance Analysis

All QC results were reported to the central office in Amsterdam, which enabled comparative monitoring of results across all programs and the identification of poorly performing laboratories. Summarized analysis was reported back to the field to enable individual laboratories to compare their performance to other laboratories in similar settings.

We use percentage agreement because it is simple, direct, and understandable at all levels [1]. Laboratory performance was considered satisfactory if the percentage agreement between the laboratory results and the reexamined results was equal to or exceeded the internal standards set by MSF-OCA (simple cut-off analysis).

## Findings

In contrast to stable programs, such as government health laboratory networks, MSF operates as an emergency humanitarian organization, and laboratory programs open and close according to changing priorities. Therefore the QC analysis presented here reflects the overall performance of MSF-OCA programs over 2005–2008 with a changing composition of laboratories.

Because only a limited number of laboratories performed leishmaniasis testing, these findings are not presented here.

To improve statistical reliability, we only analyzed percent agreement on cohort data that included at least three monthly reports in the 4-month period, and FP and FN on cohort data that included at least ten positive or ten negative slides, respectively (Table 1). Fifty-seven laboratories met these criteria for malaria microscopy QC, and 54 for TB.

**Table 1:** Laboratory QC data collection and analysis.

Data Collection and Analysis		Malaria	AFB
Total data collected	Laboratories	72	62
	Cohorts	329	325
	Monthly reports	1,093	1,074
Percent-agreement analysis (excluding cohort data that included <3 monthly reports)	Laboratories	57	54
	Cohorts	239	244
	Monthly reports	908	929
False-positive analysis (excluding cohort data that included <3 monthly reports and <10 positive slides)	Cohorts	151	177
	Monthly reports	581	675
False-negative analysis (excluding cohort data that included <3 monthly reports and <10 negative slides)	Cohorts	237	244
	Monthly reports	901	929

During the reported period, the internal MSF-OCA standards were set at ≥95% agreement for all slides (percent agreement) and ≤5% FP and FN slides.

Tests of difference between two proportions were performed using the Pearson's Chi-squared test. Analysis was performed using Epi Info 6 (US Centers for Disease Control) and STATA version 8.2 (StataCorp).

### 

#### Malaria microscopy

**Figure 1:**
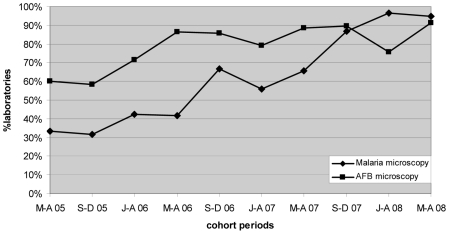
Percentage of laboratories and test centers achieving ≥95% agreement for malaria and AFB microscopy.

**Figure 2:**
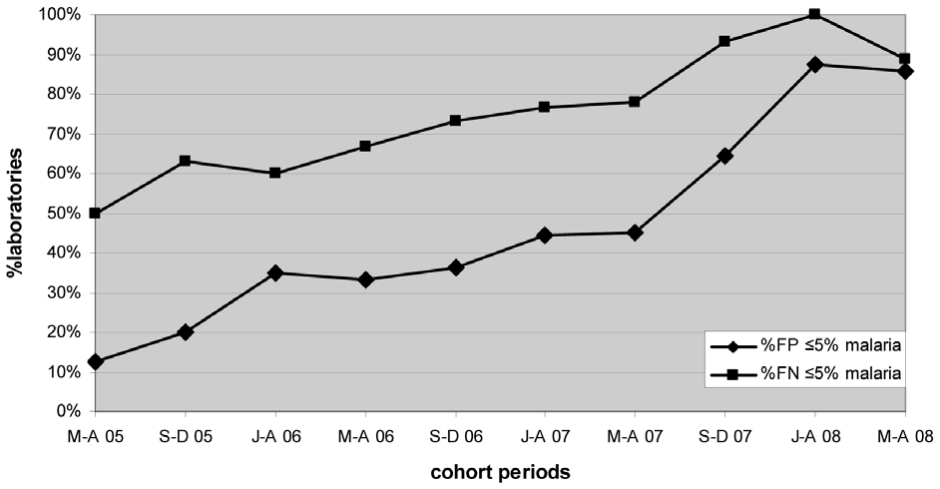
Percentage of laboratories and test centers achieving ≤5% false-positive and false-negative results for malaria microscopy.

Marked progressive improvement in the overall malaria microscopy QC performance was seen over the period (Figures 1 and 2; Table 2). At the commencement of the QC program for the period May–December 2005 (two cohorts), 32.3% (10/31), 17.4% (4/23), and 58.1% (18/31) of laboratories complied with the percent agreement, FP, and FN targets, respectively. By 2008, for the period January–August (two cohorts), there were significant improvements (*p*<0.001) in the proportion of laboratories meeting each QC target, with the results of 95.7% (45/47), 86.7% (13/15), and 91.3% (42/46), respectively.

**Table 2:** Performance of malaria and AFB microscopy.

Percentage of Laboratories and Test Centers Achieving:												
Cohort	≥95% Agreement				≤5% FP				≤5% FN			
	Malaria		AFB		Malaria		AFB		Malaria		AFB	
	Percent agreement	*n* laboratories (95% CI)	Percent agreement	*n* laboratories (95% CI)	Percent FP	*n* laboratories (95% CI)	Percent FP	*n* laboratories (95% CI)	Percent FN	*n* laboratories (95% CI)	Percent FN	*n* laboratories (95% CI)
May–Aug 05	33	4/12 (13–60)	60	6/10 (32–84)	13	1/8 (1–43)	43	3/7 (15–75)	50	6/12 (25–75)	90	9/10 (63–99)
Sep–Dec 05	32	6/19 (14–53)	58	7/12 (32–81)	20	3/15 (6–43)	44	4/9 (18–73)	63	12/19 (42–82)	67	8/12 (40–87)
Jan–Apr 06	42	11/26 (25–61)	71	15/21 (51–87)	35	7/20 (17–56)	55	11/20 (34–75)	60	15/25 (41–77)	86	18/21 (67–96)
May–Aug 06	42	10/24 (24–61)	86	19/22 (67–96)	33	7/21 (16–54)	62	13/21 (41–80)	67	16/24 (47–83)	100	22/22 (88–100)
Sep–Dec 06	67	10/15 (43–86)	86	18/21 (67–96)	37	4/11 (14–63)	64	9/14 (40–85)	73	11/15 (50–90)	86	18/21 (67–96)
Jan–Apr 07	56	19/34 (40–71)	79	23/29 (63–91)	44	12/27 (27–63)	57	13/23 (37–75)	76	26/34 (61–88)	93	27/29 (80–99)
May–Aug 07	66	21/32 (49–80)	87	31/35 (76–96)	45	9/20 (25–66)	76	19/25 (58–89)	78	25/32 (62–90)	91	32/35 (79–98)
Sep–Dec 07	87	26/30 (72–96)	90	34/38 (77–97)	64	9/14 (40–85)	83	19/23 (65–94)	93	28/30 (81–99)	95	36/38 (84–99)
Jan–Apr 08	96	27/28 (85–100)	76	25/33 (60–88)	88	7/8 (57–99)	79	15/19 (58–93)	100	26/28 (79–99)	88	29/33 (74–96)
May–Aug 08	95	18/19 (78–100)	91	21/23 (76–98)	86	6/7 (52–99)	75	12/16 (52–91)	89	16/18 (70–98)	96	22/23 (82–100)

Malaria microscopy for parasite detection, not species differentiation.

CI, confidence interval.

#### AFB microscopy

**Figure 3:**
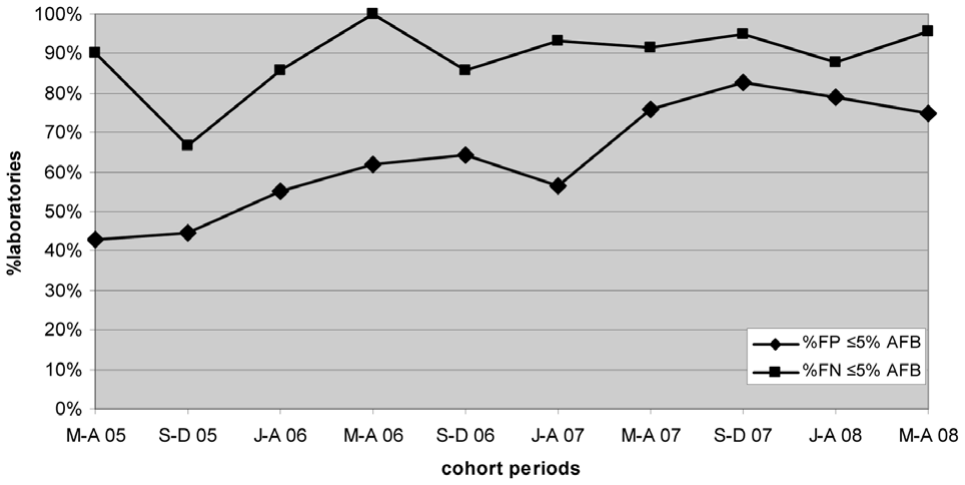
Percentage of laboratories and test centers achieving ≤5% false-positive and false-negative results for AFB microscopy.

Progressive improvement in the overall AFB microscopy QC performance was seen over the period (Figures 1 and 3; Table 2). At the commencement of the QC program for the period May–December 2005 (two cohorts), 59.1% (13/22) and 43.8% (7/16) of laboratories complied with the percent agreement and FP targets, respectively. By 2008, for the period January–August (two cohorts), 82.1% (46/56; *p* = 0.033) and 77.1% (27/35; *p* = 0.019) of laboratories, respectively, met these targets. In contrast, the FN frequency remained relatively constant throughout the period (Figure 3), with no significant difference between the May–August 2005 and May–August 2008 cohorts (90% and 96%, respectively, *p* = 0.527).

## Lessons Learned

 We found the design of our QC protocol to be practical in field settings and easily understood and implemented by laboratory staff with limited training. We attribute this to a combination of a small QC sample size, a fixed number of slides independent of the workload, and use of simple percentage agreement for statistical analysis. The small sample size considerably decreased the QC workload while maintaining statistical reliability by using targeted sampling of only weakly positive slides and 4-month cohort analysis.

Our findings show a significant improvement in the accuracy of malaria and AFB microscopy comparing the periods May–December 2005 and January–August 2008. We attribute this improvement to the strengthening of our protocols, field support, and training over this period. However our QC protocol also played a central role by providing key information on a timely basis allowing us to prioritize those laboratory support activities. Also, and we believe critically, the reporting of compiled data back to the field provided the laboratories with clear performance indicators and enabled field laboratories to directly compare their performance against other laboratories working in similar circumstances. In our experience, this generated an environment of positive “competition” among laboratories that we believe has also contributed significantly to the improvement in laboratory quality performance.

For malaria microscopy, the number of FP and FN results decreased markedly. We attribute this to active follow-up of poorly performing laboratories identified by the QC protocol. In contrast, the frequency of FN results for AFB microscopy did not change significantly, and the improvement in percentage agreement reflects the decrease in the frequency of FP results. Laboratories for AFB also entered the analysis period at a higher level of performance compared with malaria microscopy (59.1% of AFB cohorts achieving ≥95% percentage agreement for May–December 2005 compared with 32.3% for malaria). This may be because AFB microscopy is relatively easier to perform than malaria microscopy as accurate malaria microscopy requires greater microscopy resolution and has a technically more demanding staining procedure.

However we also speculate that the random selection of negative AFB smears, which is the standard methodology for AFB QC protocols and is used in our protocol, may be problematic. Saliva smears are in general more likely to be negative or have an AFB density below the threshold of microscopy detection than sputum smears [8],[9]. Therefore there is less opportunity for QC to detect FN results by reexamining saliva slides as they have a higher prior probability of being truly microscopically negative than a sputum smear. With random selection, laboratories with a high proportion of saliva samples in routine practice will also have a high proportion of saliva slides in their QC sample, and therefore the QC FN frequency for such laboratories may be lower than their true FN frequency.

For the future, we are currently incorporating clerical error monitoring into our laboratory QC protocol, as this can also be a major source of error. With the increasing emphasis on disease eradication, we are also developing QC protocols to accommodate low positivity. Finally, we have implemented a pilot study to exclude saliva smears from the AFB QC sample.

## Conclusion

From this recent field experience, our laboratory QC protocol was found to be well accepted and understood by all levels of field staff, practical in a wide variety of contexts, able to improve performance, and able to provide valuable program management information. As with all QC, implementation and sustainability requires commitment from field staff and project managers. Ongoing supervision and support are critical for central monitoring, ensuring compliance, and regular feedback reporting. The implementation of this centralized-reporting, standardized QC program has provided the catalyst for MSF-OCA to develop a laboratory “culture of quality” over the past 3 years, which in turn has strengthened the commitment and interest of laboratory field staff to ensure the success of health care programs.

## Abbreviations

AFB: acid-fast bacilli
FN: false negative
FP: false positive
LQAS: lot quality assurance sampling
MSF: Médecins Sans Frontières/Doctors Without Borders
MSF-OCA: MSF Operational Centre Amsterdam
QC: quality control
TB: tuberculosis
